# Announcement of a New Medical School in Cincinnati

**Published:** 1852-09

**Authors:** 


We have received the announcement of a new Medical School in Cincinnati, called the *Miami Medical College*.

